# Polarization of Migrating Monocytic Cells Is Independent of PI 3-Kinase Activity

**DOI:** 10.1371/journal.pone.0010159

**Published:** 2010-04-15

**Authors:** Silvia Volpe, Sylvia Thelen, Thomas Pertel, Martin J. Lohse, Marcus Thelen

**Affiliations:** 1 Institute for Research in Biomedicine, Bellinzona, Switzerland; 2 Department of Microbiology and Molecular Medicine, University of Geneva, Geneva, Switzerland; 3 Rudolf Virchow Center and Institute of Pharmacology and Toxicology, University of Würzburg, Würzburg, Germany; University Paris Sud, France

## Abstract

**Background:**

Migration of mammalian cells is a complex cell type and environment specific process. Migrating hematopoietic cells assume a rapid amoeboid like movement when exposed to gradients of chemoattractants. The underlying signaling mechanisms remain controversial with respect to localization and distribution of chemotactic receptors within the plasma membrane and the role of PI 3-kinase activity in cell polarization.

**Methodology/Principal Findings:**

We present a novel model for the investigation of human leukocyte migration. Monocytic THP-1 cells transfected with the α_2A_-adrenoceptor (α_2A_AR) display comparable signal transduction responses, such as calcium mobilization, MAP-kinase activation and chemotaxis, to the noradrenaline homlogue UK 14'304 as when stimulated with CCL2, which binds to the endogenous chemokine receptor CCR2. Time-lapse video microcopy reveals that chemotactic receptors remain evenly distributed over the plasma membrane and that their internalization is not required for migration. Measurements of intramolecular fluorescence resonance energy transfer (FRET) of α_2A_AR-YFP/CFP suggest a uniform activation of the receptors over the entire plasma membrane. Nevertheless, PI 3-kinse activation is confined to the leading edge. When reverting the gradient of chemoattractant by moving the dispensing micropipette, polarized monocytes – in contrast to neutrophils – rapidly flip their polarization axis by developing a new leading edge at the previous posterior side. Flipping of the polarization axis is accompanied by re-localization of PI-3-kinase activity to the new leading edge. However, reversal of the polarization axis occurs in the absence of PI 3-kinase activation.

**Conclusions/Significance:**

Accumulation and internalization of chemotactic receptors at the leading edge is dispensable for cell migration. Furthermore, uniformly distributed receptors allow the cells to rapidly reorient and adapt to changes in the attractant cue. Polarized monocytes, which display typical amoeboid like motility, can rapidly develop a new leading edge facing the highest chemoattractant concentration at any site of the plasma membrane, including the uropod. The process appears to be independent of PI 3-kinase activity.

## Introduction

Cell migration is an essential process for the functional positioning of cells in higher organisms. In most cases cells follow a guidance cue formed by chemoattractants that bind to specific cell surface receptors to promote chemotaxis. While the migration of tissue cells is slow and characterized by strong adhesions, leukocytes have adapted a highly motile amoeboid mechanism of migration which is in many aspects reminiscent of the amoeba *Dictyostelium discoideum* [1]. Leukocyte trafficking is a central regulatory mechanism for immune homeostasis and immune responses [2]. Upon injury neutrophils and monocytes are among the first cells leaving the blood stream to approach the site of lesion. The cells are attracted by a variety of stimuli, such as chemokines, bioactive lipids, anaphylatoxins and bacterial derived peptides, which all bind to G_i_-protein-coupled receptors (G_i_PCR). A central downstream regulatory element in receptor-mediated cell migration is the activation of phosphatidylinositide 3-kinase (PI 3-kinase) [3]–[5]. The kinase contributes, but is not mandatory to convey extracellular gradients to the intracellular organization of the responses. Furthermore, redundant pathways in chemotaxis exist for which PI 3-kinase activity is dispensable [6]–[10].

Model systems for the analysis of the signal transduction events in cells undergoing chemotaxis have provided much insight to our current knowledge on leukocyte migration. Monitoring the spatio-temporal activation of pathways has allowed refining different signaling events to the leading and trailing edge, respectively [11]. Currently few suitable systems are available that can easily be interrogated for the specific function of signal transduction components in amoeboid-like migration. Many studies were performed in *Dictyostelium* where protein expression levels can easily be altered [12], [13]. The chemotaxis of primary neutrophils and monocytes can be monitored by time laps video-microscopy, but manipulation of the expression levels of proteins in these cells is not straightforward. Most commonly neutrophil-like HL-60 cells are used to study molecular events during leukocyte migration [3], [14]–[17]. However, the cells must be differentiated to assume a functional neutrophil-like phenotype and to respond to typical agonists, such as f-Met-Leu-Phe. As a rule, however, differentiation leads to heterogeneous cell populations.

In this study we introduce the monocytic THP-1 cells to study chemotaxis. We show that cells stably transfected with the α_2A_-adrenoceptor (α_2A_AR) migrate towards the α_2A_AR agonist UK 14'304 (brimonidine). The efficacy of the chemotactic response is comparable to the stimulation with the chemokine CCL2 which binds to the endogenous CCR2. Measurements of PIP_3_ formation indicate that the cells promote the local activation of PI 3-kinase at the leading edge in response to an extracellular agonist gradient. In contrast to neutrophils and *Dictyostelium*, where the cells predominantly maintain their polarization axis and perform a U-turn in response to inversion of the chemotactic gradient, we observed that monocytes and THP-1 cells rapidly switch their polarization axis. PI 3-kinase activity rapidly relocates to what was before the uropod and after reversal of the gradient acts as leading edge. However, PIP_3_ production appears dispensable for cell polarization. Consistent with the capability of a rapid relocation of the polarization axis, receptors remain evenly distributed over the plasma membrane during cell migration.

## Materials and Methods

### Cells and cell culture

THP-1 cells (ATCC) were cultured in RPMI 1640 supplemented with 10% fetal bovine serum (FBS, HyClone), 1% glutamax, 50 U/ml of penicillin and 50 µg/ml of streptomycin (Pen/Strep, all Invitrogen).

### Transfection

Plasmids (pcDNA3) encoding for wild type α_2A_AR, α_2A_AR-YFP and α_2A_AR-YFP/CFP were as previously described [18], [19]. All receptor sequences included an HA epitope at their N-terminus. THP-1 cells were transfected by Nucleofection® (Amaxa, Lonza) according to the manufacturer's instructions. Transfected cells were grown in selection medium containing 0.7 mg/ml G418 (Invitrogen) and subcloned for highest receptor expression by FACS (FACS Aria, BD Biosciences).

### Transduction

The PH-PKB sequence [20] was subcloned in frame with a nonapeptide linker sequence (GSGGSGGSG) into pEGFP (Invitrogen) where the GFP was replaced with the sequence of the red fluorescent protein mCherry [21]. The PH-PKB-mCherry sequence was amplified by PCR using primers to incorporate *Xba1* and *Sma1* sites at the 5′ and 3′ ends (forward: 5′-AAAATCTAGAATGAACGACGTAGCCATTGTGAA; reverse 5′-TGAATTCCCGGGTTACTTGTAGAGCTCGTCCATGC). The PCR product was cloned into the pAIP-WPRE-IRES-Puro lentiviral vector [22]. Vesicular stomatitis virus-G (VSV-G)-pseudotype lentiviral vectors (LVs) were generated by co-transfecting pAIP-PH-PKB-mCherry, the HIV-1 packaging plasmid psPAX2 and the pMD2.G plasmid encoding the VSV-G envelope glycoprotein into 293T cells as described [23], [24]. Briefly, nearly confluent 293T cells in 6-well-plates were co-transfected with pALPS-PH-PKB-mCherry, psPAX2, and pMD2.G at a ratio of 4∶3∶1 (3.3 µg of total plasmid DNA) using 7.5 µl of polyethylenimine 1 mg/ml stock (MW 25000, Polysciences) 250 µL in serum-free OptiMEM medium (Invitrogen). After 24 h, the transfection medium was replaced with fresh complete RPMI medium. Forty-eight hours posttransfection, the LVs containing medium was collected and filtered through a 0.45 µm filter (Millipore). THP-1 cells cultured in 6-well plates were treated with 2 ml of VLP. Positive clones were selected by FACS.

### FACS analysis of receptor expression

THP-1 cells (200 µl containing 10^5^cells) were incubated with 200 nM CCL2 [20] or 1µM UK 14'304 at 37°C for the indicated times. Incubations were terminated by the addition of ice cold PBS and washing of the cells. The cells were stained for CCR2 with 5 µg/ml anti CCR2 (MAB150 R&D System) or 5 µg/ml anti HA (12CA5, Roche) for 30 min on ice, washed and incubated with 10 µg/ml goat anti-mouse IgG-RPE (Southern Biotech). Isotype control was performed with 5 µg/ml mouse IgG2_2b_ (Southern Biotech).

### Calcium and Chemotaxis

Intracellular free calcium was measured as previously described [25]. Chemotaxis assays were performed in triplicate in 48-well Boyden chambers (NeuroProbe, Gaithersburg, MD), using 5µm pore-sized polyvinylpyrrolidone-free polycarbonate membranes. Chemotaxis medium (RPMI-1640 supplemented with 1% FBS, 1% glutamax, Pen/Strep) containing chemoattractants was added to the lower wells. Cells (10^5^ per well) in chemotaxis medium were added to the upper wells and incubated for 40 min at 37°C in 5% CO_2_ atmosphere. Cells were removed from the upper part of the membrane with a rubber policeman. Cells attached to the lower side of the membrane were fixed and stained. Migrated cells were counted in five randomly selected fields of 100-fold magnification. Where indicated, cells were pretreated at 37°C for 2 h with 2 µg/ml *Bordetella pertussis* toxin (Sigma).

### Western blot analysis

The cells were starved for 6 h in serum-free RPMI 1640 medium supplemented with 2% bovine serum albumin (BSA, Sigma). When indicated pertussis toxin 2 µg/ml (Sigma) was added to the medium. Cells were washed once in phosphate buffered saline (PBS) and stimulated at 37°C for 2 min with UK 14'304 300 nM and CCL2 100 nM. Incubations were terminated by the addition of ice-cold trichloro-acetic acid. Processing of cell lysates and ERK phosphorylation was performed as described [20].

### Microscopy

Receptor internalization was determined on fixed permeabilized specimens as follows. THP-1 cells were plated on poly-D-lysine coated coverslips in medium. Following adherence the cells were stimulated for 30 min at 37°C. Cells were washed with warm PBS and then fixed with 4% paraformaldehyde on PBS for 20 min at room temperature. Cells were washed again with PBS, the reaction quenched with 100 mM glycine in PBS (20 min), and permeabilized for 5 min with PBS containing 0.1% (w/v) TritonX-100 (Sigma). Cells were washed with PBS containing 0.02% Tween 20 (w/v) (PBST) and incubated with primary rabbit anti-CCR2 (Sigma) for one hour. Following washing with PBST the cells were incubated with secondary anti-mouse- or anti-goat-IgG conjugated with Alexa488 (Invitrogen), DAPI and phalloidin-Alexa594 (Invitrogen). Coverslips were mounted as previously described [20].

Laser scanning confocal microscopy was performed with a Leica DI6000 microscope stand connected to a SP5 scan head equipped with a temperature controlled chamber (Cube, LIS, Basel). Live cell cultures were placed in a humidified and CO_2_-controlled incubator which was mounted on the microscope stage (Brick, LIS, Basel).

For time-lapse video microscopy cells (0.5×10^6^/ml) were resuspended in D-PBS containing calcium and magnesium (Invitrogen) supplemented with 1% FBS, Pen/Strep, 0.04 mM sodium pyruvate, 1 mg/ml fatty acid free BSA (Sigma), 1 mg/ml glucose (Fluka). Cells were plated on glass bottom petri-dishes (MatTek cultureware) which were coated before with D-poly-lysine (5 µg/ml) and subsequently overlaid with a 1∶80 diluted Matrigel® (BD Biosciences) solution at 4°C for 30 min.

Chemoattractants were dispersed with a micropipette (Femtotip II, Eppendorf) controlled by micromanipulator (Eppendorf) at a constant backpressure of 15 hPa (Femtojet, Eppendorf).

For CFP/YFP FRET measurements cells were excited with a UV laser (405 nm) at low power setting (∼15%), and the emission of CFP (F_CFP_ = 465 nm-505 nm) and the FRET signal (YFP emission, F_YFP_ = 525 nm–600 nm) were measured contemporaneously. Ratio FRET (rFRET) was calculated as FRET/F_CFP_ (F_YFP_/F_CFP_) as described [26] using the Metamorph software package (Visitron). Due to the low laser intensity used for excitation fading of CFP and YFP was usually <5% over 1 min.

## Results

**Figure 1:**
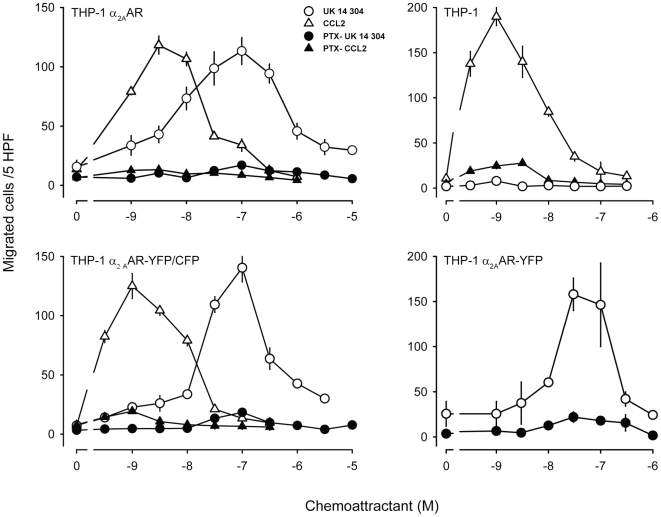
Chemotaxis of THP-1 cells mediated by endogenous and transfected receptors. Either wild type (upper right) or transfected cells (panel labeling denotes expressed receptor) were subjected to chemotaxis assays in modified Boyden chambers for 40 min. Chemotaxis was stimulated with UK 14'304 (circles) or CCL2 (triangles) without (open symbols) or with pretreatment with pertussis toxin (closed symbols).

Non-chemokine pertussis toxin-sensitive G_i_-protein coupled receptors can mediate chemotaxis [27]. Therefore we reasoned that the G_i_-protein-coupled α_2A_-adrenergic receptor (α_2A_AR), when expressed in the migration competent THP-1 cells, could induce cell migration. The α_2A_AR belongs to the group of adrenoceptors that transduce responses to catecholamines during neurotransmission and is known to mediate hypotension, sedation and analgesia [28], but it is not known to stimulate cell migration. THP-1 cells were stably transfected with the α_2A_AR and tested in chemotaxis assays using modified Boyden chambers. Figure 1 shows the migratory behavior of wild type (top right) and transfected (top left) THP-1 cells in response to the chemokine CCL2, an agonist of the endogenous CCR2, and to UK 14'304, an agonist with high affinity and specificity for α_2A_AR [29]. In THP-1 cells expressing α_2A_AR (top left) CCL2 and UK 14'304 induced chemotaxis with similar efficacy, albeit with different efficiencies. The differences in efficiency was unexpected because both agonist possess comparable affinities for their respective receptors (Kd ∼2–3 nM CCL2/CCR2; Kd ∼2 nM UK 14'304/α_2A_AR) [29]–[31]. A maximum of the typical bell shape migratory response for CCL2 was obtained between 1–3 nM, whereas the UK 14'304 stimulated response peaked at 100 nM. Wild type THP-1 cells did not respond to UK 14'304 (top right), indicating that chemotaxis observed in the transfected cells was entirely dependent on the newly introduced α_2A_AR. Pertussis toxin pretreatment fully abrogated cell migration in response to CCL2 and UK 14'304, confirming that both receptors couple to G_i_-proteins.

For the study of receptor activity and localization in polarized migrating cells we took advantage of α_2A_AR variants which were tagged with yellow fluorescent protein (YFP) at the C-terminus (THP-1 α_2A_AR-YFP) or with yellow fluorescent protein (YFP) at the third intracellular loop and CFP at the C-terminus (THP-1 α_2A_AR-YFP/CFP). Both constructs were previously characterized for ligand binding and response properties [18], [19], [32]. After stably transfecting the constructs in THP-1 cells, we tested if the tagged receptors, which bear a considerable increase in molecular mass, were competent in mediating chemotaxis. Figure 1 (bottom right) shows that fusion of YFP to the C-terminus of α_2A_AR had no major effect on UK 14'304 stimulated chemotaxis. Similarly, the response to CCL2 was also not affected (not shown). Even more critical for receptor function could be the incorporation of two fluorescent proteins. However, THP-1 cells expressing α_2A_AR-YFP/CFP showed almost identical chemotactic responses to UK 14'304 and CCL2 as THP-1 α_2A_AR cells (Figure 1 left panels). Thus, the inclusion of the tags does not appear to compromise coupling of the receptor to downstream elements responsible for mediating cell migration. Moreover signaling via G_iα,_ which requires the interaction of the G-protein with the second and third intracellular loop, appears to be preserved, because the response was abolished by pertussis toxin pretreatment.

To continuously monitor migration of THP-1 cells, time-lapse video microscopy was established. Glass bottom culture dishes coated with a monolayer of poly-Lys were incubated with a diluted Matrigel® solution at 4°C for 30 min. Excess of Matrigel® was removed and the dishes rinsed with PBS. Because Matrigel® does not polymerize at low temperatures, the procedure leads to a monolayer like coating of the dishes allowing observation of cell migration at high resolution in a single optical plane. Among different coatings tested, such as fibrinogen, fibronectin and collagen, coating with Matrigel® appeared to provide the optimal substratum to support THP-1 migration. The chemoattractants CCL2 or UK 14'304 were released from a micropipette driven by constant backpressure. Movie S1 shows typical migration sequences of wild type and THP-1 cells expressing the double tagged α_2A_AR-YFP/CFP in response to UK 14'304 and CCL2. Both agents attracted the cells towards the tip of the dispensing pipette and moving the pipette reoriented the migration of the cells.

**Figure 2:**
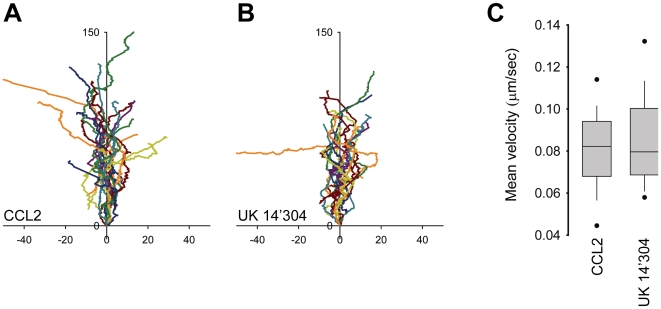
Velocity of THP-1 cells migrating towards different agonists. THP-1 α_2A_AR-YFP/CFP cells were plated on glass bottom coverslips coated with poly-D-lysine and Matrigel®. (A) CCL2 (10 nM) and (B) UK 14'304 (1 µM) were dispensed from a micropipette with constant backpressure. Time-lapse videos for each conditions were recorded at 5 sec interval. Tracks were analyzed and plotted aligning their average directional vector with the y-axis (distance in µm). (C) Mean velocity of tracks shown in (A) and (B) were calculated using Metamorph. Data from two independent observations, n = 17 (A) and n = 20 (B).

Analysis of tracks recorded by time-lapse video microscopy from multiple cells stimulated with 10 nM CCL2 or 1 µM UK14'304 indicated that migration towards the chemokine was somewhat more efficient (Figure 2A and B), despite of similar velocity of the movement (Figure 2C). The migratory index (MI), i.e. the distance between the starting point and the end point divided by the total migrated distance, was 0.704±0.15 (SD) for CCL2 and 0.614±0.17 (SD) for UK 14'304. The mean velocity of THP-1 cell migration on matrigel coated glass appears to be slightly slower than that of mouse neutrophils migrating in EZ-Taxiscan chambers on fibrinogen coated glass, 0.08 µm/sec vs. 0.11 µm/sec, respectively [10]. Overall the velocity of migration and the MI of THP-1 cells are only moderately less efficient compared with parameters reported for primary mouse neutrophils migrating in EZ-Taxiscan chambers on fibrinogen coated glass [10].

**Figure 3:**
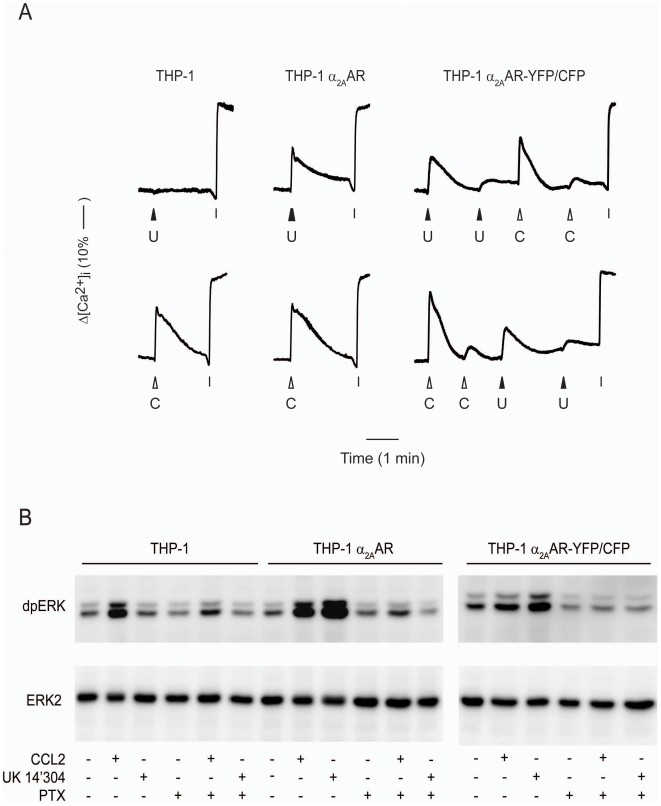
THP-1 cells-mediated signal transduction by endogenous and transfected receptors. (A) Intracellular calcium mobilization. Wild type and transfected THP-1 cells were loaded with Fura-2 and stimulated with 300 nM UK 14'304 (U) or 100 nM CCL2 (C). Fluorescence signals were normalized to maximum calcium influx elicited by the addition of ionomycin (I) as described in Methods. (B) Activation of ERK1/2 in THP-1 cells. Wild type and transfected THP-1 cells non-treated or pretreated with pertussis toxin (PTX) were stimulated for 2 min with 100 nM CCL2 or 300 nM UK14'304. Upper images are Western blots of dual phosphorylated ERK1 and 2, below total ERK2 (loading reference).

Next we investigated the ability of α_2A_AR transfected THP-1 cells to trigger changes in intracellular free calcium [Ca^2+^]_i_. Figure 3A (lower left) shows a typical rise in [Ca^2+^]_i_ upon stimulation of wild type THP-1 cells with CCL2, but as expected from the chemotaxis experiments no response was obtained with UK 14'304 (upper left). In THP-1 α_2A_AR cells, CCL2 stimulated a similar response, but in addition these cells also displayed a marked rise in [Ca^2+^]_i_ following stimulation with UK 14'304. Also cells expressing the doubly tagged receptor THP-1 α_2A_AR-YFP/CFP exhibited typical calcium elevations in response to CCL2 and UK 14'304 (right panels). When cells were exposed sequentially to either agonist, an almost complete desensitization to a second bolus of the same agonist was observed, but the response to the other stimulus remained normal. Thus, neither CCL2 nor UK 14'304 cross-desensitized each other indicating that the two receptors function autonomously. Following treatment of the cells with pertussis toxin, calcium elevations with either stimulus were abolished (not shown).

Rapid phosphorylation of the MAPK kinases ERK1/2 following stimulation with chemokines is amply reported as a measure of receptor-mediated cell activation. We used Western blot analysis to detect dual phosphorylated ERK1/2 (dpERK) and show that in THP-1 cells CCL2 induced a marked activation of ERK1/2 (Figure 3B, lane 2). The addition of UK 14'304 to wild type THP-1 had no effect, further confirming that the cells do not express endogenous functional receptors for this agonist. However, THP-1 α_2A_AR displayed a marked activation of ERK2 in response to CCL2 and to UK 14'304 (Figure 3B lane 8 and 9). Similarly, THP-1 α_2A_AR-YFP/CFP responded to both agonists with a marked activation of ERK1/2 (right panel). In all instances phosphorylation of ERK1/2 was prevented if the cells were pretreated with pertussis toxin, confirming that the receptors couple to heterotrimeric G_i_-proteins. Taken together these findings indicate that α_2A_AR and its tagged variants when expressed in THP-1 cells induce similar responses as endogenous chemokine receptors.

**Figure 4:**
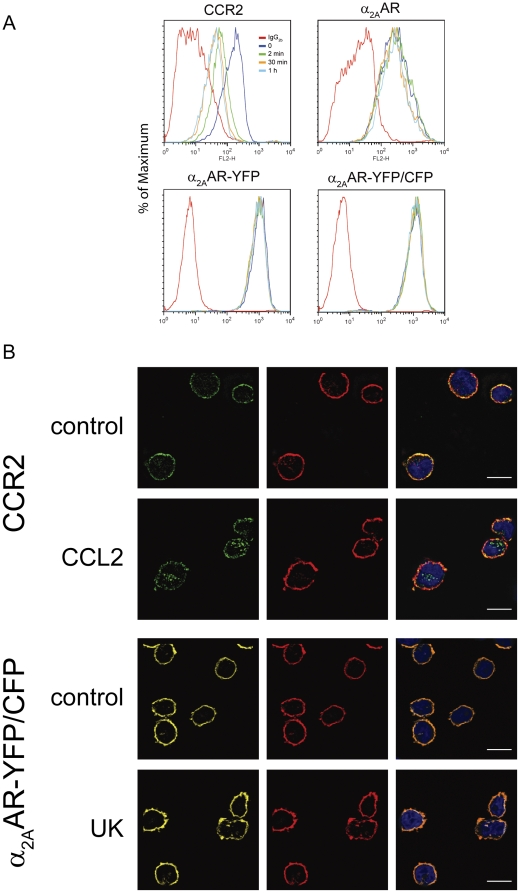
Receptor internalization. (A) FACS analysis. THP-1 cells were incubated with 200 nM CCL2 and CCR2 expression was measured after the indicated times (upper left). Similarly, THP-1 cells stably transfected with α_2A_AR (upper right), α_2A_AR-YFP and α_2A_AR-YFP/CFP (lower panels) were incubated with 1µM UK 14'304. Red lines represent isotype controls (IgG_2b_). (B) Confocal microscopy images of wild type THP-1 cells (upper panels) and THP-1 transfected with α_2A_AR-YFP/CFP (lower panels). Cells were untreated (control) or stimulated with 200 nM CCL2 or 1µM UK14'304 (UK) for 30 min fixed and processed as described in Methods. Receptor expression in wild type (CCR2) was revealed with specific anti-CCR2 (left panel, green). For localization of α_2A_AR-YFP/CFP the fluorescence of receptor associated YFP was measured (left panel, yellow). The middle panels depict F-actin revealed with phalloidin-Alexa594. Right panels are merged images including nuclear staining (DAPI, blue).

In general GPCR internalize following agonist stimulation via clathrin-mediated endocytosis [33]. However, the necessity of receptor internalization during cell migration remains controversial. Several reports indicate that internalization of chemokine receptors is required for chemotaxis [34]–[37], while others provide evidence that internalization is not required [27] or show that lack of internalization can enhance chemokine-mediated cell migration [38]. Stimulation of THP-1 cells with CCL2 resulted in a time-dependent down regulation of CCR2 (Figure 4A). By contrast, the α_2A_AR, either wild type or tagged with fluorescent proteins, was not down regulated from the cell surface even after extended treatment with UK 14'304. Receptor internalization was measured by FACS analysis using the mAb 12CA5 which detects the HA epitope at the N-terminus of the α_2A_AR constructs. Similarly, confocal microscopy revealed refractoriness of α_2A_AR-YFP/CFP to become internalized following stimulation, whereas upon activation with CCL2 CCR2 was readily detected on endosomal structures (Figure 4B). Our observation that UK 14'304 does not induce major internalization of α_2A_AR in the THP-1 cells mirrors findings obtained with the non-hematopoietic HEK293 and COS-1 cells transfected with α_2A_AR [39]–[42]. Together these data indicate that α_2A_AR-YFP/CFP-mediated chemotaxis, calcium mobilization and ERK2 phosphorylation, which are comparable with the responses elicited by the endogenous CCR2, do not depend on receptor internalization.

**Figure 5:**
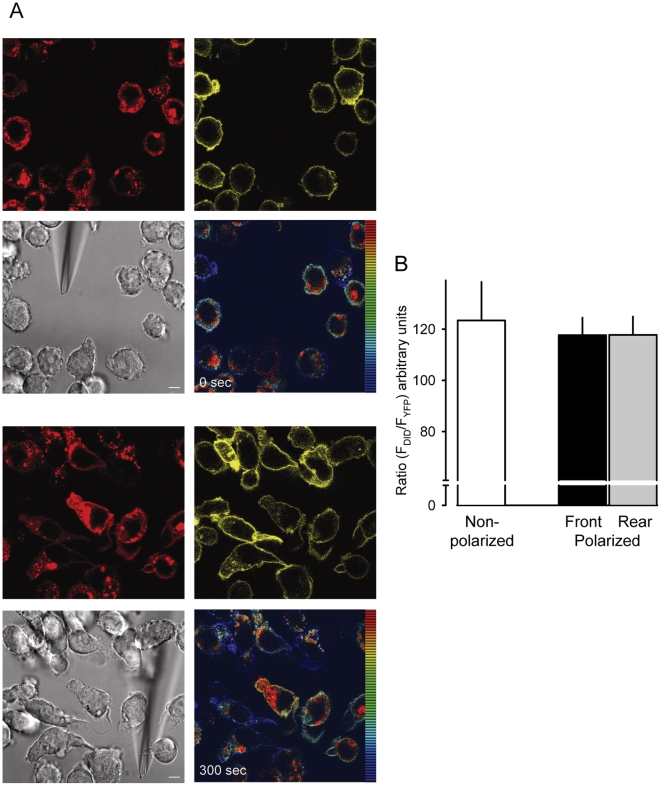
Receptor localization in resting and polarized cells. (A) Selected frames from Movies S2 just after insertion of the micropipette dispensing 300 nM UK 14'304 (0 sec, upper images) and after 5 min (300 sec, lower images). Red fluorescence derives from the membrane marker DiD (594 nm excitation/620–680 nm emission), yellow fluorescence derives from the YFP tag of α_2A_AR CFP/YFP (514 nm excitation/525–590 nm emission). Both fluorescences were recorded contemporaneously. The false color shows the ratio of red/yellow. Gray, corresponding phase image. The bar in the phase images represents 5 µm. (B) Average ratio-intensity analysis of regions of interest (ROI) measured in resting cells (white bar, non polarized) and polarized cells stimulated with 300 nM UK 14'304 at the front (black) or rear (grey). Data represent the mean values from multiple frames from 3 different cells each from 3 independent experiments. The threshold for data inclusion was set equal in all experiments.

The localization of chemotaxis-mediating receptors on the plasma membrane of leukocytes is still contentious. While studies on lymphocytes conclude that receptors are recruited to the leading edge and accumulate at the immunological synapse [43]–[48], others have shown that in amoebae and neutrophil like cells (PBL-985, HL-60) the receptors remain evenly distributed over the plasma membrane of polarized cells [49]–[52]. We used the fluorescently tagged receptors to address the question whether the GPCRs distribute uniformly at the plasma membrane in polarized monocytic cells. THP-1 cells expressing α_2A_AR-YFP/CFP were labeled with the Vybrant DiD cell-labeling solution (DiD) and then subjected to time-lapse video microscopy. Figure 5A shows representative confocal frames at time 0 and after 5 min from movie S2 of cells migrating in response to UK 14'304 taken with high optical resolution (63x) and a narrow pinhole at the confocal unit. Over the total observation period, the (red) dye appeared evenly associated with the plasma membrane and accumulated in intracellular organelles. Similarly, the fluorescence of YFP seemed uniformly distributed over the entire plasma membrane in the confocal images. Ratiometric analysis of DiD/YFP fluorescence intensity shows a homogeneous color marking over the entire plasma membrane in resting and migrating cells. The observation is consistent with the concentration of membrane marker and receptors being constant and suggests that the ratio does not alter when cells polarize and migrate. The quantitative analysis from multiple cells of the DiD/YFP fluorescence intensity ratio of the leading edge and the rear confirms the conclusion (Figure 5B). Thus, the constant ratio over the entire membrane illustrates that the receptors do not re-localize to the membrane of the leading edge in THP-1 cells.

**Figure 6:**
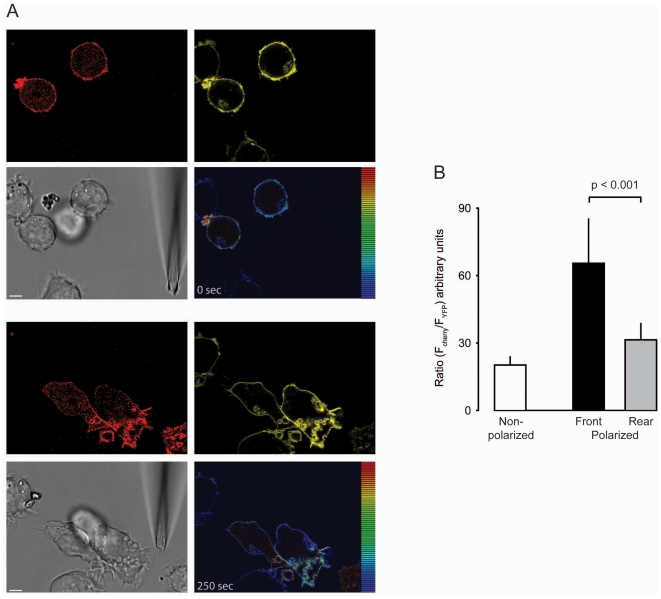
PIP_3_ formation occurs exclusively at the leading edge. (A) Selected frames from Movies S3 just after insertion of the micropipette dispensing 300 nM UK 14'304 (0 sec, upper images) and after 5 min (250 sec, lower images). The emission of THP-1 cells transduced with PH-PKB-mCherry (red fluorescence, 594 nm excitation/610–680 nm emission) and expressing α_2A_AR-YFP/CFP (yellow fluorescence YFP, 514 nm excitation/525–580 nm emission) was recorded contemporaneously (right panels). The false color shows the ratio of red/yellow. Gray, corresponding phase image. The bar in the phase images represents 5 µm. (B) Average ratio-intensity analysis of regions of interest (ROI) measured in resting cells (white bar, non polarized) and polarized cells stimulated with 300 nM UK 14'304 at the front (black) or rear (grey). Data represent the mean values from multiple frames from 3 different cells each from 3 independent experiments. The threshold for data inclusion was set equal in all experiments.

Despite the even distribution of chemoattractant receptors in polarized cells in amoebae and leukocytes, local activation of PI 3-kinase occurs solely at the leading edge [4], [53], [54]. We tested the activation of PI 3-kinase in THP-1 cells stably transformed with a reporter construct specific for the kinase product phosphatidylinositol (3,4,5) triphosphate (PIP_3_) [53]. The construct consisted of the PH-domain of protein kinase B (PKB) fused to a short linker sequence and cloned in frame to the N-terminus of the red fluorescent protein mCherry (PH-PKB-mCherry). THP-1 cells expressing the α_2A_AR-YFP/CFP and the PH-PKB construct were analyzed by time-lapse video microscopy. Unstimulated cells revealed a moderate red fluorescence in the cytoplasm and at the plasma membrane. When the cells started to polarize and migrated towards the micropipette dispensing UK 14'304 a pronounced accumulation of the reporter construct at the leading edge was observed (Figure 6A and movie S3). Given that the α_2A_AR-YFP/CFP does not alter its distribution during migration (Figure 5B), the YFP fluorescence was used to normalize the PH-PKB-mCherry fluorescence intensity per voxel. Figure 6A and movie S3 shows a marked increase of the mCherry fluorescence intensity with respect to the YFP signal of the receptor consistent with a pronounced PIP_3_ formation at the leading edge. The asymmetric distribution of the PKB-PH domain further indicates that PI 3-kinase is almost exclusively activated at the leading edge and that the PIP_3_ formation occurs concomitantly with the cell becoming polarized (movie S3). When α_2A_AR-YFP/CFP and PH-PKB-mCherry expressing THP-1 cells were stimulated with CCL2 a similar strong activation of PI 3-kinase at the leading edge was observed (not shown). Figure 6B reports the mean fluorescence intensity in resting non-polarized cells and in UK 14'304-induced polarized cells at the front and the rear. The analysis indicates an average two-fold increase in PIP_3_ after 30-40 sec at the leading edge compared to the rear (Figure 6B).

**Figure 7:**
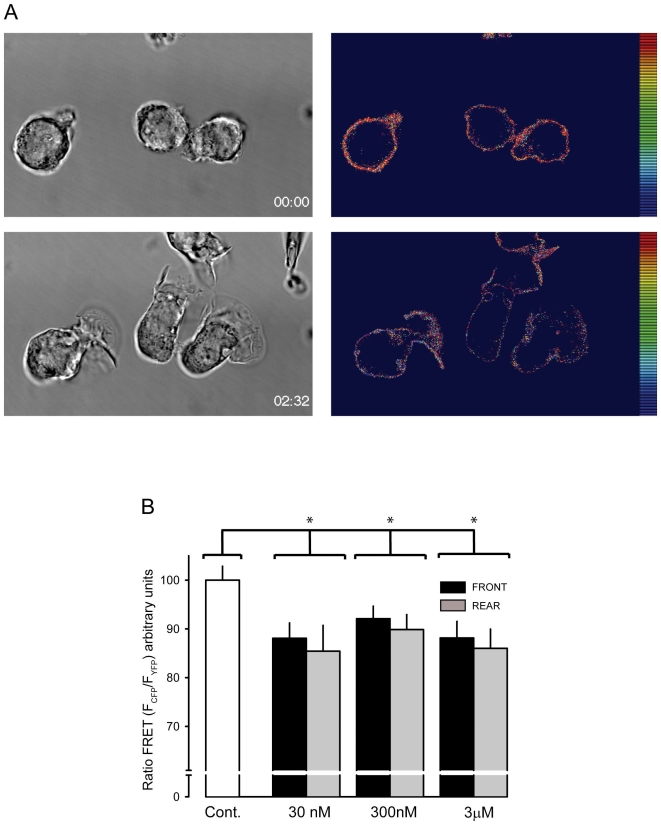
FRET efficiency of α_2A_AR-YFP/CFP in migrating cells. (A) Selected frames from Movies S4 just after insertion of the micropipette dispensing 300 nM UK 14'304 (0 sec, upper images) and after 152 sec (2:32, lower images). Ratio FRET of THP-1 cells expressing α_2A_AR-YFP/CFP was measured as described in Methods. The false color shows FRET efficiencies. Gray, corresponding phase image. (B) Quantitative analysis of FRET efficiencies at the beginning (Cont., white bar) and 2–3 min after stimulation measured at the front (black bar) and rear (grey bar). Mean values of 3 cells from 3 independent experiments (30 nM and 300 nM UK 14'304) and 3 cells from 2 experiments for 3 µM UK 14'304. Stars indicate statistical relevance of p<0.0001 (T-test).

The above observations suggest that receptor-mediated signal transduction leading to PI 3-kinase activation occurs by and large at the leading edge. However, the gradient which is formed by the dispensing pipette should also be sensed at the back of the cells albeit at up to 30% lower concentration. The exact chemoattractant concentration over the cell is difficult to determine in this experimental set up. The flow rate from the micropipette cannot accurately be determined due to the non-linear flow conditions imposed by the narrow orifice of the pipette and the free diffusion of the ligand. However, cell migration can be induced with a wide range of concentrations of the filling solution, such as 30 nM–3 µM UK 14'304 or CCL2 with similar efficiencies. Given the dissociation constant of UK 14'304 for α_2A_AR-YFP/CFP being 3–4 nM [19] and an estimated concentration difference between the front and the rear of a migrating cell of <30%, this indicates that at any of the above conditions the concentration of the attractant at the rear of the cells is sufficiently high to occupy the receptors. Taking these reflections into account it is unclear why PIP_3_ production occurs only at the leading edge. It has been proposed that at the rear of a polarized cell the signal transduction from GPCRs is offset at the Gi-protein level through a global inhibitory process [55]. To test receptor activity at the front and the rear we took advantage of the intramolecular FRET of the α_2A_AR-YFP/CFP as an indicator. The stoichiometry of the donor (CFP) and acceptor (YFP) of the construct is constant, therefore the FRET signal is independent of the local concentration of the receptor [26], [56]. Ligand-induced α_2A_AR-YFP/CFP activation causes a conformational change leading to the reduction of the FRET efficiency between CFP and YFP [19]. The maximum change in FRET efficiency measured as normalized ratio FRET (F_YFP_/F_CFP_) of the α_2A_AR-YFP/CFP expressed in THP-1 exposed to 1 µM UK 14'304 was ∼10%, similar to the changes reported in HEK293 cells [19]. Figure 7A depicts frames from a time lapse-video at time 0 and 152 sec of THP-1 α_2A_AR-YFP/CFP cells stimulated with 300 nM UK 14'304 from a pipette. At both times the FRET efficiency is continuous over the entire plasma membrane. However, markedly different ratios were observed, showing a high FRET efficiency at time 0 and a reduced efficiency after 152 sec (false color scale) indicating homogeneous receptor activation over the entire membrane. Movie S4 displays the complete sequence of the gradual change in FRET efficiency over time. Importantly, although a clear net change in FRET was obtained, we observed at no time a difference in FRET efficiency between the leading edge and the rear of the cells. A quantitative analysis of the FRET efficiency measured at the front and the rear of polarized cells exposed for 2 min to 30 nM, 300 nM and 3 µM UK 14'304 is shown in Figure 7B. The data reveal within the limits of the accuracy of the measurements that there was no significant difference in FRET efficiency between the leading edge and the posterior side.

**Figure 8:**
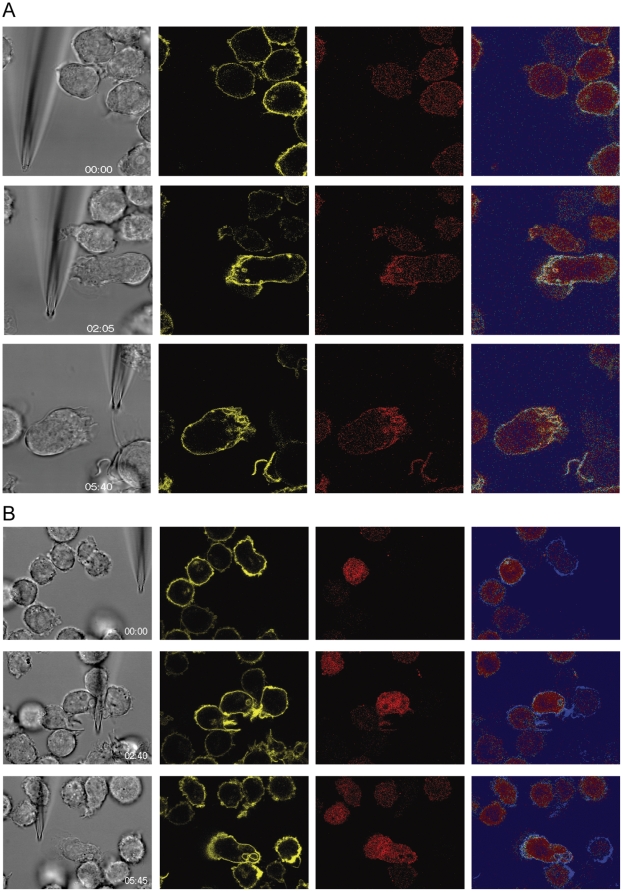
Role of PIP_3_ formation during reversal of polarization. Selected frames from Movies S6 just after insertion of the micropipette dispensing 300 nM UK 14'304 (0 sec, upper images) and after 5:40 min (lower images). The emission of THP-1 cells transduced with PH-PKB-mCherry (red fluorescence, 594 nm excitation/610–680 nm emission) and expressing α_2A_AR-YFP/CFP (yellow fluorescence YFP, 514 nm excitation/525–580 nm emission) was recorded contemporaneously. The false color shows the ratio of red/yellow (right panels). Gray, corresponding phase image. (A) control cells, (B) cell pretreated for 10 min with 100 nM Wortmannin. (514 nm excitation/525–590 nm emission), (Phase) phase images taken with DIC settings, and (Ratio) False color shows the ratio of red/yellow.

Amoeboid migrating cells, such as neutrophils and *Dictyostelium*, once polarized in a gradient, largely maintain their polarization axis [54], [57]. *Dictyostelium* when stimulated with a relatively large concentration of cAMP (100 µM) or upon rapid reversal of flow-induced hydrodynamic shear stress can, however, reverse its polarization axis [58], [59]. The vast majority of neutrophils upon reversal of the gradient reorient their leading edge to the source of the gradient by performing U-turns [4], [54], [57]. However, a small fraction (up to 20%) can revert their polarization [60]. Movie S5 illustrates the typical behavior of most neutrophils stimulated with 10 µM f-Met-Leu-Phe. By contrast, almost all freshly isolated human monocytes fully reverse their polarization axis in less than one minute upon reversal of the gradient produced by 100 nM CCL2. Moreover, movie S5 demonstrates that the polarization axis could be switched several times. Similar results were obtained with THP-1 cells (movie S6 and S7). Figure 8A depicts selected frames from the Movie S6 which demonstrate that PI 3-kinase activity upon the reversal of the polarization axis re-localized to the new leading edge (right panels). Interestingly, reversal of the polarization axis observed in the phase images appears to precede the activation of PI 3-kinase at the new leading edge. When the experiment was performed in the presence of 100 nM Wortmannin, the cells were able to migrate toward the dispensing pipette and to flip their polarization axis following displacement of the source of attractant (movie S7). Moreover, neither polarization nor reversal of the axis was associated with PI3-kinase activation. The frames from the movie shown in Figure 8B illustrate that the PKB-PH domain probe in the presence of Wortmannin does not localize at the leading edge, but rather remains diffuse in the cytoplasm. These observations suggest that receptor-mediated activation of PI 3-kinase is not required for monocyte polarization. The observation is consistent with a number of publications that have contested a general requirement of PI 3-kinase activation in cell migration [6], [9], [10], [61]–[64].

## Discussion

Only few model systems for the study of leukocyte migration are currently available. The here described monocytic THP-1 cells bear some advantage over the classical neutrophil-like HL-60 cells. THP-1 can easily be transfected or virally transduced and do not need to be differentiated to reach a chemotaxis-competent phenotype. The relatively homogenous cell populations of wild type or transformed cells uniformly respond to ligands of endogenous chemotactic receptors such as CCL2 (CCR2) or CXCL12 (CXCR4, not shown). In addition the cells are capable to migrate in response to stimulation of ectopically expressed receptors such as the α_2A_AR. Interestingly the highly conserved DRY(L/I)AI(V/I) motif of chemokine receptors that is considered critical for chemoattractant-induced and G_i_-protein-mediated cell migration is only partially conserved in the *DRY*WSIT sequence of the α_2A_AR. Nevertheless the present data demonstrate that the structural properties of the α_2A_AR suffice to mediate efficient chemokine receptor-like signal transduction and cellular responses in THP-1 cells stimulated with the α_2A_AR agonist UK 14'304.

Following global stimulation most chemokine receptors internalize and become down regulated by an β-arrestin depended pathway [65], [65,66]. In THP-1 cells CCR2 follows this paradigm; upon stimulation its appearance at the plasma membrane becomes punctuated, consistent with ligand-induced lateral segregation of the receptor, as initial step in clathrin-mediated endocytosis leading to the localization of the receptor in large endosomal structures [67]. By contrast, neither wild type nor fluorescent protein tagged α_2A_AR expressed on THP-1 cells appear to segregate or internalize. Previous studies in COS-1 cells indicated that α_2A_AR requires β-arrestin2 for internalization [41]. Our observation indicates that for efficient migration receptor internalization might be dispensable. However, we can not exclude that during migration at the leading edge, rapid recycling of the receptors may occur, which does not go beyond the formation of very early endosomes and does not lead to fusion with large endosomes [68]. Such early endosomes must remain close to the plasma membrane and can therefore not be visualized by confocal microscopy.

Our data provide strong evidence that chemotactic receptors in THP-1 cells remain uniformly distributed in the plasma membranes and do not accumulate in the membrane forming the leading edge. In polarized cells we observed a net increase in plasma membrane towards the front of the cells which is caused by ruffling and lamellipodia formation. Macroscopically this would indicate a relative increase in receptor density at the front versus the sides and the rear of the cells where the membrane appears to be more smooth. As a consequence, through a feedback mechanism more signal transduction events could be engaged at the front leading the stabilization of the polarization axis. However, the fact that the polarization axis of monocytic cells can rapidly reverse impinges on the model. In movie S5 we show the repeated rapid reversal of the polarization axis in monocytes which were attracted with 10 µM CCL2. Thus, the reorientation initiates at a site with markedly lower membrane density suggesting that the relative receptor density may not be sufficient to maintain the polarization axis in monocytes. Furthermore, flipping of the polarization was observed with different agonist concentrations (30 nM–10 µM). In Movie S5 we applied 10 µM CCL2, a concentration sufficiently high to saturate CCR2 at the front and presumably also at the rear. Considering the slow dissociation rate of the CCL2 from the CCR2 (half life >200 min) [69], it is conceivable that the receptors remain ligated during the entire recording period. Taken together, the observations suggest that chemotactic receptors presumably sense small differences in the relative agonist concentration rather than the absolute amount [52]. The conclusion is consistent with our FRET-based receptor activation measurements and would predict that differences in receptor activity are minimal and below the detection limit of the probe.

Extracellular gradients are assumed to translate into steep intracellular ramps of enzymatic reactions. Local activation of PI 3-kinase at the leading edge has been proposed as hallmark of polarized cells [54], [62]. Several downstream effectors which are directly or indirectly regulated by 3-phosphoinositides and are involved in chemotaxis have been identified [3], [4]. In agreement with these observations we show that PI 3-kinase is rapidly activated during THP-1 cell polarization where PIP_3_ accumulation is restricted to the leading edge. The two-fold increase is probably a cautious estimate considering the basal membrane association of the reporter construct in non-polarized cells (Figure 6B). The absence of PIP_3_ at the posterior end of the cell may reflect several points of interference with agonist-stimulated PI 3-kinase activation. Besides the potential direct inhibition of any signal transduction step leading to PIP_3_ formation local activation of phosphatases such as PTEN or SHIP-1 could account for the absence. Both phosphatases have been implicated in restricting PIP_3_ to the leading edge and preventing the lateral diffusion [70], [71]. It was also proposed that in polarized cells feedback loops enhance PIP_3_ formation at the leading edge even in the absence of receptor activation [3], [62]. On the other hand time-lapse video microscopy showed that efficient chemotaxis can occur in the absence of PI 3-kinase activation [9]. It was proposed that PI 3-kinase activity is involved in integrin mediated neutrophil adhesion and F-action accumulation at the leading edge [10]. Here we provide evidence (Movie S6) that cells can migrate towards the dispensing pipette and that polarization occurs in the absence of the activation of PI 3-kinase. Moreover, in the presence of wortmannin the polarization axis can be reversed further underlining that polarization *per se* is independent of PI 3-kinase activity.

## Supporting Information

Movie S1 CCR2-mediated migration of THP-1 α_2A_AR-YFP/CFP cells. Cells were plated on glass bottom coverslips coated with poly-D-lysine and Matrigel®. CCL2 (100nM) was dispensed from a micropipette (center) with constant backpressure. Time-lapse video was recorded at 6 sec interval with DIC optics at 20× magnification. In the second part the cells were stimulated with 1 µM UK 14'304.
(9.18 MB WMV)

Movie S2 Receptor localization in resting and polarized cells. Frames were taken at 4 sec interval with a 63× magnification. (DiD) Red fluorescence derives from the membrane marker DiD (594 nm excitation/620–680 nm emission), (YFP) yellow fluorescence derives from the YFP tag of α_2A_AR-YFP/CFP (514 nm excitation/525–590 nm emission), (Phase) phase images taken with DIC settings, and (Ratio) False color shows the ratio of red/yellow.
(7.71 MB WMV)

Movie S3 PIP_3_ formation occurs exclusively at the leading edge. Frames were taken at 5 sec interval with a 63× magnification. (PH) Red fluorescence derives from PH-PKB-mCherrry probe which binds to PIP_3_, (YFP) yellow fluorescence derives from the YFP tag of α_2A_AR-YFP/CFP (514 nm excitation/525–590 nm emission), (Phase) phase images taken with DIC settings, and (Ratio) False color shows the ratio of red/yellow.
(5.94 MB WMV)

Movie S4 FRET efficiency of α_2A_AR-YFP/CFP in migrating cells. Frames were taken from THP-1 cells expressing α_2A_AR-YFP/CFP and stimulated with 1 µM UK 14'304 at 4 sec interval with a 63× magnification. (FRET YFP) FRET signal (YFP emission, FYFP = 525 nm–600 nm), (CFP) CFP emission (FCFP = 465 nm–505 nm), (PHASE) phase images taken with DIC settings, and (Ratio FRET) Ratio FRET (rFRET) was calculated as FRET/FCFP (FYFP/FCFP). The false color shows FRET efficiencies.
(4.68 MB WMV)

Movie S5 Migration of freshly isolated human neutrophils and monocytes. Neutrophils were prepared as previously described [64], frames were taken at 3 sec interval with DIC optics. Cells were stimulated with 10 µM f-Met-Leu-Phe. Monocytes were prepared as described [65] were taken at 3 sec interval with DIC optics. Cells were stimulated with 100 nM CCL2.
(10.01 MB WMV)

Movie S6 Role of PIP_3_ formation during reversal of polarization. Frames were taken at 5 sec interval with a 63× magnification from control THP-1 α_2A_AR-YFP/CFP cells stimulated with 300 nM UK 14'304. (PH) Red fluorescence derives from PH-PKB-mCherrry probe which binds to PIP_3_, (YFP) yellow fluorescence derives from the YFP tag of α_2A_AR-YFP/CFP (514 nm excitation/525–590 nm emission), (Phase) phase images taken with DIC settings, and (Ratio) False color shows the ratio of red/yellow.
(6.46 MB WMV)

Movie S7 Role of PIP_3_ formation during reversal of polarization. Frames were taken at 5 sec interval with a 63× magnification from THP-1 α_2A_AAR-YFP/CFP cells pretreated for 10 min with 100 nM Wortmannin and stimulated with 300 nM UK 14'304. (PH) Red fluorescence derives from PH-PKB-mCherrry probe which binds to PIP_3_, (YFP) yellow fluorescence derives from the YFP tag of α_2A_AR-YFP/CFP (514 nm excitation/525–590 nm emission), (Phase) phase images taken with DIC settings, and (Ratio) False color shows the ratio of red/yellow.
(6.31 MB WMV)
